# Effectiveness of a virtual intervention for primary healthcare professionals aimed at improving attitudes towards the empowerment of patients with chronic diseases: study protocol for a cluster randomized controlled trial (e-MPODERA project)

**DOI:** 10.1186/s13063-017-2232-9

**Published:** 2017-10-30

**Authors:** Ana Isabel González-González, Carola Orrego, Lilisbeth Perestelo-Perez, Carlos Jesús Bermejo-Caja, Nuria Mora, Débora Koatz, Marta Ballester, Tasmania del Pino, Jeannet Pérez-Ramos, Ana Toledo-Chavarri, Noemí Robles, Francisco Javier Pérez-Rivas, Ana Belén Ramírez-Puerta, Yolanda Canellas-Criado, Yolanda del Rey-Granado, Marcos José Muñoz-Balsa, Beatriz Becerril-Rojas, David Rodríguez-Morales, Luis Sánchez-Perruca, José Ramón Vázquez, Armando Aguirre

**Affiliations:** 1 Centro de Salud Vicente Muzas, Gerencia Asistencial de Atención Primaria, Servicio Madrileño de Salud, Red de Investigación en Servicios de Salud en Enfermedades Crónicas (REDISSEC), Calle Vicente Muzas 8, 28043 Madrid, Spain; 2 Instituto Universitario Avedis Donabedian, Universidad Autónoma de Barcelona, Red de Investigación en Servicios de Salud en Enfermedades Crónicas (REDISSEC), Calle Provença 293 pral, 08037 Barcelona, Spain; 3 Servicio de Evaluación y Planificación, Dirección del Servicio Canario de la Salud, Red de Investigación en Servicios de Salud en Enfermedades Crónicas (REDISSEC), Camino Candelaria s/n. Centro de Salud El Chorrillo, 38109 El Rosario, Santa Cruz de Tenerife, Spain; 4 Unidad de Apoyo Técnico, Gerencia Asistencial de Atención Primaria, Servicio Madrileño de Salud, Calle San Martín de Porres 6, 28035 Madrid, Spain; 5 Fundación Canaria de Investigación y Salud, Hospital Universitario de Canarias, Pl. -1. Crta. La Cuesta-Taco, 38320 La Laguna, Tenerife Spain; 6 Agència de Qualitat i Avaluació Sanitàries de Catalunya (AQuAS), Red de Investigación en Servicios de Salud en Enfermedades Crónicas (REDISSEC), Calle Roc Boronat 81-95, 08005 Barcelona, Spain; 7 Dirección Técnica de Procesos y Calidad, Gerencia Asistencial de Atención Primaria, Servicio Madrileño de Salud, Facultad de Enfermería de la Universidad Complutense de Madrid, Calle San Martín de Porres 6, 28035 Madrid, Spain; 8 Centro de Salud Vicente Muzas, Gerencia Asistencial de Atención Primaria, Servicio Madrileño de Salud, Calle Vicente Muzas 8, 28043 Madrid, Spain; 9 Centro de Salud Monóvar, Gerencia Asistencial de Atención Primaria, Servicio Madrileño de Salud, Calle Monóvar 11, 28033 Madrid, Spain; 10 Área de Cronicidad, Subdirección General de Continuidad Asistencial, Servicio Madrileño de Salud, Calle San Martín de Porres 6, 28035 Madrid, Spain; 11 Dirección Técnica de Sistemas de Información Sanitaria, Gerencia Asistencial de Atención Primaria, Servicio Madrileño de Salud, Red de Investigación en Servicios de Salud en Enfermedades Crónicas (REDISSEC), Calle San Martín de Porres 6, 28035 Madrid, Spain; 12 Gerencia de Atención Primaria de Tenerife del Servicio Canario de la Salud, Calle Monteverde 45, 38003 Tenerife, Spain; 13 Hospital Universitario Nuestra Señora de la Candelaria, Ctra. Gral. del Rosario 145, 38010 Tenerife, Spain

**Keywords:** Empowerment, Primary healthcare, Virtual system, Healthcare professional attitudes

## Abstract

**Background:**

Communities of practice are based on the idea that learning involves a group of people exchanging experiences and knowledge. The e-MPODERA project aims to assess the effectiveness of a virtual community of practice aimed at improving primary healthcare professional attitudes to the empowerment of patients with chronic diseases.

**Methods:**

This paper describes the protocol for a cluster randomized controlled trial. We will randomly assign 18 primary-care practices per participating region of Spain (Catalonia, Madrid and Canary Islands) to a virtual community of practice or to usual training. The primary-care practice will be the randomization unit and the primary healthcare professional will be the unit of analysis. We will need a sample of 270 primary healthcare professionals (general practitioners and nurses) and 1382 patients. We will perform randomization after professionals and patients are selected. We will ask the intervention group to participate for 12 months in a virtual community of practice based on a web 2.0 platform. We will measure the primary outcome using the Patient-Provider Orientation Scale questionnaire administered at baseline and after 12 months. Secondary outcomes will be the sociodemographic characteristics of health professionals, sociodemographic and clinical characteristics of patients, the Patient Activation Measure questionnaire for patient activation and outcomes regarding use of the virtual community of practice. We will calculate a linear mixed-effects regression to estimate the effect of participating in the virtual community of practice.

**Discussion:**

This cluster randomized controlled trial will show whether a virtual intervention for primary healthcare professionals improves attitudes to the empowerment of patients with chronic diseases.

**Trial registration:**

ClicalTrials.gov, NCT02757781. Registered on 25 April 2016.

**Protocol Version**. PI15.01 22 January 2016.

**Electronic supplementary material:**

The online version of this article (doi:10.1186/s13063-017-2232-9) contains supplementary material, which is available to authorized users.

## Background

Increased life expectancy has contributed to the growing prevalence of chronic diseases. Different European studies report that chronic diseases affect more than 80% of people aged over 65 years, are responsible for 86% of deaths and generate premature morbidity and loss of years of healthy life [1]. The growing burden of these diseases also has massive economic consequences, ranging from the impoverishment of individuals and families to a considerable increase in healthcare system costs and the potential weakening of economies [2]. It has been estimated that 70–80% of healthcare costs in Europe are currently allocated to chronic disease management [3].

These important epidemiological changes are pushing multiple healthcare reforms across Europe, aimed at enhancing sustainability and transforming healthcare systems — from a focus on acute-care needs to a focus on chronic and multiple diseases. A key trend in these reform processes is the inclusion of the key concept of patient empowerment [4].

Although there is no single generally accepted definition of patient empowerment, most definitions focus on the ability of individuals to make decisions about their health (behaviour) and to take control of health-related aspects of their lives [5, 6].

A recently proposed definition in the European context is that used for the EMPATHIE project, led by the Instituto Universitario Avedis Donabedian [7]: An empowered patient has control over the management of their disease on a daily basis, acts to improve their quality of life and has the necessary knowledge, skills, attitudes and perceptions to adjust their behaviour — and work in partnership with others when necessary — to achieve optimal wellness. Interventions aimed at empowerment aim to equip patients (or their informal caregivers, as appropriate) with the ability to participate in decisions about their illness to the degree they wish, to become co-managers of their disease in partnership with healthcare professionals, and to develop self-confidence, self-esteem and the skills necessary to deal with the physical, emotional and social impact of the disease on their daily lives.

There are a number of prerequisites for patient empowerment, but two of the most important are a certain level of health literacy and the support of professionals [8], given that the attitude of professionals is widely acknowledged as a key variable in achieving patient empowerment [9]. More patient-centred healthcare professionals are not only more likely to empower patients managing chronic diseases [10, 11], but also to enhance patient satisfaction and improve adherence to treatments [12–14].

The Eurobarometer Qualitative Study on Patients Involvement [15] found that most healthcare professionals consider themselves to be primarily responsible for the health of their patients. Only when directly asked did they concede that patients also held some responsibility, in that they could adopt healthy lifestyles or preventive measures, provide information to assist diagnoses and comply with the instructions of healthcare professionals. Most professionals felt that patient involvement improves care quality by helping patients feel more motivated to take better care of themselves, better understand their disease and its treatment and better monitor their health independently. Healthcare professionals need to increase their awareness of patients’ capacities to assume a more active role in their own care and to learn mechanisms that increase this capacity.

The community of practice (COP) was described in 1991 by Lave and Wenger [16] as a “group of people who share an interest, a set of problems or a passion about a topic, and who deepen their knowledge and experience in the area through continuous interaction that strengthens relationships.”

In COPs, therefore, which are based on the concept of lifelong learning, learning involves exchanges of experiences and knowledge between people. COPs differ from conventional working groups in several ways [17]: (1) member participation is voluntary and reflects a common interest; (2) COPs are informal, but although there is no hierarchy, they do have a structure; (3) learning is viewed as a process of participation and shared leadership; and (4) COPs are flexible and generally interconnect with other groups within or outside the organization. Several authors have written about the positive impact of COPs in improving the quality of care [17–19]. In the healthcare environment, active social interaction, knowledge sharing, knowledge creation and the construction of identity by COP members are, as in other similar environments, key elements [20, 21].

Although COPs as a means of learning are still relatively novel in health care [16], since 2005 there has been a notable increase in publications on the topic [22–26], although the scientific evaluation of their results is not systematic. A common problem is the lack of baseline and comprehensive longitudinal tracking. To our knowledge, no clinical trial testing the effectiveness of virtual communities for healthcare professionals has been published to date.

This project proposes an approach with a triple benefit: increasing knowledge of empowerment among professionals, harnessing the potential benefit of COPs as an innovative educational tool and rigorous assessment of the instrument e-MPODERA.

**Fig. 1:**
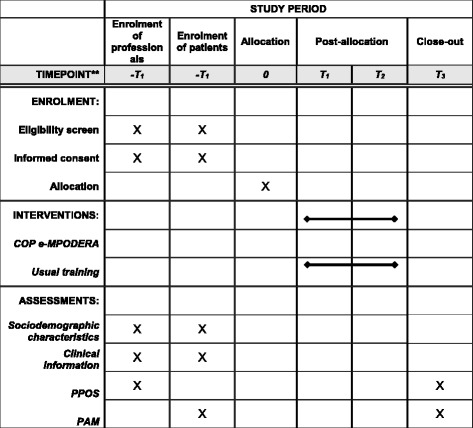
Standard Protocol Items: Recommendations for Interventional Trials (SPIRIT) study timeline

This protocol has been prepared in accordance with the Standard Protocol Items: Recommendations for Interventional Trials (SPIRIT) checklist and figure (Additional file 1 and Fig. 1).

## Aim

This article describes the design of a project called Effectiveness of a Virtual Intervention to Improve Healthcare Professional Attitudes to Patient Empowerment (e-MPODERA). Our main aim was to evaluate the effectiveness of a COP in improving attitudes of primary healthcare professionals to the empowerment of patients with chronic diseases, as measured using the Patient Practitioner Orientation Scale (PPOS) questionnaire [27]. As a secondary aim we also evaluated the effectiveness of a COP in improving the activation of patients with chronic diseases, as measured using the Patient Activation Measure (PAM) questionnaire [28]. We hypothesize that a virtual COP intervention will improve primary healthcare professional attitudes to empowering patients with chronic diseases.

## Methods

### Design

This is a multicentre pragmatic clinical trial, with two parallel arms comparing the intervention with usual training, with randomized allocation by clusters.

### Setting

The setting is in primary-care practices (PCPs) — clusters — from three regions of Spain (Catalonia, Madrid and the Canary Islands).

### Participants

#### PCPs (clusters)

Urban or rural PCPs located in Catalonia, Madrid or the Canary Islands, willing to participate and with adequate Internet connectivity that enables access to the COP.

#### Primary healthcare professionals

The primary healthcare professionals involved in the study will be general practitioners (GPs) and nurses working in the selected PCPs who volunteer to participate in the study after signing informed consent. Healthcare professionals who participate in the study should have no intention of moving from their practice during the study period.

#### Patients

Inclusion criteria are: (1) patients aged 18 years and older; (2) patients whose electronic medical records include an active diagnosis, made at least one year prior to inclusion in the study, of any of the following chronic diseases: hypertension, diabetes mellitus, hypercholesterolaemia, obesity, heart failure, ischaemic heart disease, cerebrovascular disease, chronic renal disease, chronic obstructive pulmonary disease or asthma; (3) patients who have consulted their GP or nurse about their chronic disease at least twice in the previous 12 months; and (4) patients who have signed the informed consent for participation in the study. Exclusion criteria are: (1) patients unlikely to cooperate with the study; (2) patients with a temporary residency; (3) patients who are institutionalized; (4) patients with terminal illnesses; (5) patients with a physical or mental disability that prevents them from responding suitably to questionnaires; and (6) patients whose telephone or email details for later contact are not in the PCP dataset.

### Sample size

We calculated the required number of primary healthcare professionals for the purpose of comparing two independent means, considering as significant a post-intervention mean difference of 0.2 points and a common standard deviation of 0.5 points in the PPOS questionnaire between the e-MPODERA virtual COP intervention and control groups [29]. Each group will need 100 professionals, assuming alpha error of 0.05 and power of 80%. Since we will randomize by clusters, we have adjusted the sample size to take into account the design effect. Considering an intraclass correlation coefficient of 0.03 and assuming an average size per cluster of five healthcare professionals, we obtained a design effect of 1.12 [30]. Given these assumptions and expecting 20% loss to follow up after 12 months, we calculated a sample size of 270 healthcare professionals (135 professionals per group). The recruited healthcare professionals will, in turn, recruit a total of 1382 patients (assuming 20% loss to follow-up), the number that is required to detect a mean difference between the two groups of 4 points in the Patient Activation Measure questionnaire (PAM) scale [28].

### Recruitment

Table 1 depicts the various stages of the e-MPODERA trial.

**Table 1:** e-MPODERA randomized controlled trial stages

Stage	Procedure	
Stage 1 1^st^ month	Face-to-face or virtual meetings with the selected PCPs in each of the 3 Spanish regions. Invitation to participate. Participation agreement form signed by the PCP director	
Stage 2 1^st^ month	Face-to-face or virtual meetings in each selected PCP. Invitation to participate. Informed consent form signed by professionals who agree to participate	
Stage 3 2^nd^ month	PAM questionnaire administration to all eligible patients (baseline)	
Stage 4 2^nd^ month	PPOS questionnaire administration to all included professionals (baseline)	
Stage 5 2^nd^ month	PCP randomization	
Stage E 6 2-13^th^ month	Intervention group Virtual community of practice	Control group Usual training
Stage 7 14^th^ month	PPOS questionnaire administration to all included professionals (post intervention)	
Stage 8 14 − 15^th^ month	PAM questionnaire administration to all eligible patients (post intervention)	

*PCP* Primary-care practice, *PAM* Patient Activation Measure, *PPOS* Patient-Practitioner Orientation Scale

#### PCP recruitment

From the total pool of PCPs in Catalonia (329), Madrid (262) and the Canary Islands (102), we will randomly select 18 PCPs from each participating region (total 54 PCPs) using computer-generated random tables. We will contact the selected PCPs by e-mail/telephone to briefly explain the study and to request their participation and will subsequently provide a more detailed explanation of the study in face-to-face or virtual meetings of PCP directors or designated professionals. We will randomly assign PCPs that agree to participate to either the e-MPODERA virtual COP intervention group or the control (usual training) group. Before randomization we will request PCP directors to sign a participation agreement.

#### Professional and patient recruitment

We will hold face-to-face or virtual meetings with GPs and nurses from each selected PCP. Before PCP randomization, all included professionals (at least one per cluster) will complete both an informed consent form (Additional file 2) and the PPOS questionnaire, so as to minimize possible information bias. Also before PCP randomization, to avoid possible selection bias, the participating healthcare professionals will consecutively include patients. Patients who meet the inclusion criteria will complete, in their first visit, both an informed consent form (Additional file 3) and the PAM questionnaire.

### Randomization

**Fig. 2:**
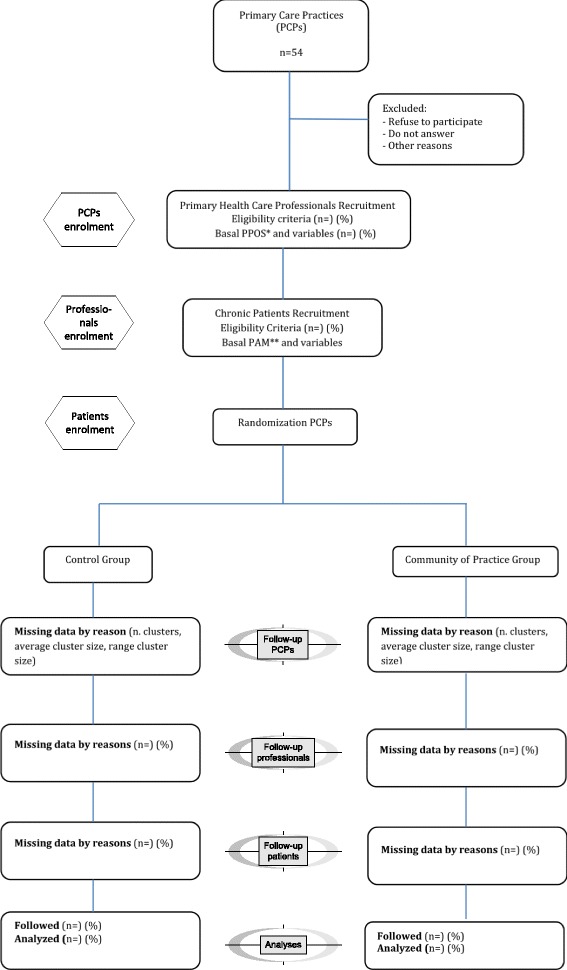
Randomization flowchart. *Patient-Practitioner Orientation Scale questionnaire **Patient Activation Measure questionnaire

We will perform allocation by clusters — with the PCP as the randomization unit — to minimize possible contamination effects among professionals. An investigator blinded to PCP identity and to baseline PPOS and PAM results will centrally perform randomization after professionals and patients are selected. This investigator will randomly assign 54 PCPs (nine pairs for each of the three regions, each randomly assigned a code) to either the e-MPODERA virtual COP group or the control group (27 PCPs per group) using Epidat 4.1 software (balanced group option). The unit of analysis will be the primary healthcare professional. To reach the necessary sample size, each professional will be required to recruit at least six consecutive patients. Figure 2 depicts a flowchart of the randomization procedure.

### Masking

It is not possible to mask the intervention in a study of this nature. Patients and the persons in charge of data analysis will be blind to the group to which each PCP will be assigned.

### Intervention

#### Control group

Professionals in the control PCPs will follow the standard training referred to in the training plans of the region where the PCP is located. The healthcare services for these regions provide ongoing training in the form of face-to-face group courses or online individual courses for healthcare professionals charged with the care of patients with chronic diseases.

#### e-MPODERA virtual COP intervention group

Professionals in the PCPs assigned to the virtual COP group will receive a link via email to register and start voluntarily participating in the e-MPODERA COP. e-MPODERA is a virtual knowledge-sharing COP based on a web 2.0 platform (http://dev.epract.net/community2/empodera). It will feature educational and gamified elements — including lectures, articles, newsletters, a resource bank, forums and virtual (video/teleconferencing) meetings — aimed at facilitating learning and transferring knowledge and experiences between participants. All researchers in the e-MPODERA group will be involved in developing the COP by providing updated content.

The platform structure and components will aim to respond to the needs and interests of professionals through a customized itinerary and different levels of depth (approaches to challenges, problem solving, cooperation among participants, feedback). The intervention will propose that participants identify and apply tools and knowledge models and a moderator/facilitator (community manager) will ensure proper functioning of the platform. To design activities, a competence framework will be used that covers core competences and includes learning objectives. We will progressively include 3 thematic areas related to patient empowerment: health literacy, self-care (health knowledge, self-efficacy, behavioural change, lifestyle change, sign/symptom monitoring, technical skills, acceptance of the chronic disease) and shared decision-making (autonomy and self-determination). We will address the following topics: (1) strategies and tools to empower patients with chronic diseases (rating scales, diagnostic tools and improving ability to understand patient information); (2) the impact on outcomes of empowering patients; (3) experiences and best practices of empowerment; and (4) barriers and facilitators, methods and approach models.

### Outcome measures

#### Primary outcome

##### Healthcare professional attitudes

Specific measurement: the PPOS questionnaire (Additional file 4), developed by Krupat et al. in 1999 [27] will be used: this is a validated self-administered questionnaire containing 18 items scored on a 6-point Likert scale (1–6) that has been demonstrated to have very good psychometric properties [12, 27]. Professionals are classified by the PPOS into 3 categories according to their patient-centeredness score: low (≤4.57), medium (>4.57 and < 5), or high (≥5) [27]. We will translate and adapt the PPOS instrument for Spanish using the forward and back-translation procedure and will validate it within the e-MPODERA trial.

Specific metrics: change from baseline

Methods of aggregation: we will measure the mean of the PPOS questionnaire score. Time points: we will measure the primary outcome at the individual level by administering the PPOS questionnaire at baseline and at 12 months.

#### Secondary outcome

##### Patient level of activation

Specific measurement: we will assess the patient level of activation with the PAM activation questionnaire (Additional file 5). The PAM activation questionnaire, which assesses activation in patients with chronic diseases, is the most widely used tool to measure patient engagement in self-care [31]. A short version of 13 items developed by Hibbard in 2005 [28] evaluates patient beliefs, knowledge and confidence in terms of a wide range of health-related behaviours. It uses a 4-point Likert-type scale (1–4) with a total score that is translated into a scale ranging from 0 to 100 (100 is the highest level of activation) to make interpretation easier. A version has been translated into Spanish and has been validated [32]. We will use the Insignia Health PAM® Survey (http://www.insigniahealth.com/products/pam-survey) in this project.

Specific metrics: we will assess change from baseline. Methods of aggregation: we will measure the mean of the PAM questionnaire score. Time points: we will measure the secondary outcome at the individual level by administering the PAM questionnaire at baseline and at 12 months.

### Additional measures

We will collect relevant demographic and medical data at baseline from the patients themselves or from the medical record and other administrative registers: (1) for primary healthcare professionals: age, sex, PCP, Spanish region, and professional profile (speciality, care setting and years of professional experience); and (2) for patients: type and duration (years) of the primary chronic disease, number of chronic diseases, age, sex, education level (illiterate, primary incomplete, primary complete, secondary complete or university), occupation, marital status (married, single, divorced or widowed), living circumstances (alone or accompanied), level of Spanish language comprehension (low, medium or high), and healthcare resource use (number of GP or nurse visits in the past year).

### Data collection, management and analyses

We will collect a mixture of professional and patient-reported data, using validated questionnaires for the primary and most of the secondary outcome measures. We will require each recruiting site to keep accurate and verifiable source notes regarding each participant’s inclusion and continued participation in the study. We will collect, transfer and store data in accordance with Good Clinical Practice (GCP) guidelines and data protection requirements. The e-MPODERA standard operating procedures (SOPs) and study data management plan will define the exact procedures for collecting, transferring, storing and quality-controlling study data.

We will perform descriptive and exploratory analyses, including cluster analyses according to participant baseline characteristics, using means and standard deviations for continuous variables and proportions for categorical variables. We will compare the two groups in terms of PPOS scores, PAM scores and descriptive variables. We will perform inferential analysis using the Mann-Whitney U test — with a 95% confidence interval — to compare across-group results in the PPOS and PAM tests in terms of score at 12 months from baseline.

We will perform a multilevel analysis of models for the main outcome (the PPOS test), where the response variable will be the difference between initial and final scores in the questionnaire administered to participating professionals and the explanatory variable will be the dichotomous intervention variable (1 = COP/0 = control). This model considers covariates such as age, sex and professional profile and also takes into account the random effect associated with the COP. We will use the propensity score to control for possible selection bias and enhance the accuracy of the model.

We will perform intention-to-treat analyses using the statistical software (http://www.R-project.org/) and PASW Statistics 18. We will use the last observation carried forward to impute missing data and will report analyses with and without imputation. We will inform participants that they can leave the study at any time. We will report missing data, if applicable, by categorizing the participants as follows: mistakenly randomized, did not receive the intervention, withdrew consent, crossed over, dropped out, did not adhere, lost contact or other reasons not specified [33].

### Study timeline

Trial start: January 2017.

Start of baseline data collection and intervention in general practice: March 2017.

End of intervention in general practice: March 2018.

End of 12-month follow-up data collection: May 2018.

Start of data analysis: June 2018.

Planned study end date: December 2018.

Duration: 2 years.

### Trial organization

CO will have overall responsibility for day-to-day trial management and for the trial in Catalonia and will also be the head of the Trial Management Group. AIGG and CJBC will have overall responsibility for the trial in Madrid. LP will have overall responsibility for the trial in the Canary Islands. NM will act as the trial statistician. The Trial Management Group will meet once a month throughout the study to ensure that all trial activities are organized according to the protocol and within the timescales set out in the original application for funding.

### Harm

The intervention is safe although we will closely monitor unintended consequences through the e-MPODERA platform with the help of the moderator. Risks arising from participation are considered to be very low. The proposed interventions are already offered to patients throughout Spain and internationally. The only difference conferred by participation is that the intervention will be randomly allocated and more carefully assessed.

## Discussion

Conceptual and practical approaches to patient empowerment have developed exponentially over the past decade and reflect a shift away from paternalistic perceptions of the professional-patient relationship and towards an interactional-model approach centred in equity and collaboration, according to the bioethical principle of autonomy [34].

In this study we will assess whether participation in the e-MPODERA virtual COP improves the attitudes of primary healthcare professionals to the empowerment of patients with chronic diseases. We will also assess whether this change in the attitude of healthcare professionals will enhance patient empowerment.

### Study strengths and limitations

Currently — and despite the importance of maintaining the knowledge and skills of healthcare professionals — conventional approaches to continuing professional development (lectures, meetings) seem insufficient to translate learning to practice [35, 36]. We will experimentally test an innovative learning intervention based on a COP in the field of patient empowerment, for which the literature lacks experimental evaluations. This project, furthermore, proposes a COP in a virtual format, using technology to facilitate communication between COP members in different geographic locations and even different time zones, thereby increasing the diversity of the network [37].

A virtual COP offers theoretical and tangible benefits for improving professional knowledge and implementation in clinical practice [37], as follows:

- It is a unique, dynamic, semi-structured system set within an environment of continuous learning and offering immediate access to a broad range of knowledge.
- It offers access to repositories of current or historical discussions (archived knowledge) and enables access to peer or senior professionals regarding the application of appropriate knowledge in practice.
- It acts as a common platform for the discussion and exchange of ideas and resources (documents, data, audio files, videos, etc) aimed at ensuring optimal patient care.

Many training activities in the healthcare sector are currently designed for only one discipline or profession and so do not address the need for the perspective of inter-professional teams practising in these systems or settings [38–40]. In the experience of healthcare professionals, it appears that interacting with peers fosters learning and information sharing [16]. The e-MPODERA project will facilitate such inter-professional interactions.

We will run the clinical trial in three regions that are broadly representative of the Spanish national healthcare system, with publicly funded care models and different healthcare management models. Primary healthcare professionals across Spain — mainly GPs and nurses — have similar curricula (in terms of degrees and diplomas) and the GP specialization is based on similar training programmes. During the trial we will only allow the selected PCPs access to the COP, so as to limit the “viral” potential of the COP. Opening a second phase of open-access COP will control this limitation.

In similar experiences [41], the participation rate has been low, with just 30% of professionals accepting the invitation and only 3% becoming active in the COP. Therefore, we calculated the data sample so as to reduce the risk of a low participation rate that would render it difficult to attribute any change in PPOS and PAM scores to the e-MPODERA intervention. To enhance participation in the COP, we will implement several strategies, including an active role by a community manager, weekly emails as reminders that summarize the most important issues of the week with relevant information attached and a competitive score system according to the goals achieved.

## Trial status

Recruitment commenced in November 2016 and is ongoing at the time of submission.

## Additional files

Additional file 1: SPIRIT (Standard Protocol Items: Recommendations for Interventional Trials) checklist. (DOC 121 kb)

Additional file 2: Informed consent for primary healthcare professionals. (DOC 90 kb)

Additional file 3: Informed consent for patients. (DOC 90 kb)

Additional file 4: Patient-Practitioner Orientation Scale (PPOS) questionnaire. (DOCX 16 kb)

Additional file 5: Patient Activation Measure (PAM) questionnaire. (DOCX 16 kb)

## Abbreviations

COP: Community of practice
e-MPODERA: Effectiveness of a Virtual Intervention to Improve Healthcare Professional Attitudes to Patient Empowerment
GCP: Good Clinical Practice
GP: General practitioner
PAM: Patient Activation Measure
PCP: Primary-care practice
PI: Principal investigator
PPOS: Patient-Practitioner Orientation Scale
SOP: Standard operating procedure
