# Supplementary material for: Effectiveness of a virtual intervention for primary healthcare professionals aimed at improving attitudes towards the empowerment of patients with chronic diseases: study protocol for a cluster randomized controlled trial (e-MPODERA project)
Source: Trials. 2017 Oct 30;18:505. doi: 10.1186/s13063-017-2232-9 (PMC5663036; doi:10.1186/s13063-017-2232-9)
Supplement: Supplementary file 2 — Informed consent for primary healthcare professionals. (DOC 90 kb) [file 13063_2017_2232_MOESM2_ESM.doc]

**Additional file 2**

**CONSENTIMIENTO INFORMADO**

**PROFESIONALES**

Estudio: “Evaluación de la efectividad de una intervención (Comunidad de Práctica virtual) dirigida a profesionales para mejorar su actitud hacia el empoderamiento de pacientes con enfermedades crónicas: Ensayo controlado aleatorizado por conglomerados” (Cataluña: PI15/00164, Madrid: PI15/00586, Canarias: PI15/00566)

Yo, __________________________________________________________________Nombre y apellidos del profesional sanitario

· He leído la información que se me ha entregado.

· He recibido suficiente información sobre el estudio.

· Comprendo que mi participación es voluntaria.

· Comprendo que puedo negarme a participar o retirarme del estudio cuando lo desee, sin tener que dar explicaciones y sin que perjudique a mi persona o trabajo.

Con la siguiente firma, **ACEPTO** participar en el estudio.

Firma del profesional sanitario:

Firma del investigador:

**Datos de contacto del profesional:**

Teléfono: Correo electrónico:

Con la siguiente firma, **NO ACEPTO** participar en el estudio.

¿Por qué?:

Actividad profesional en AP (Años):

Firma del profesional sanitario: Firma del investigador:

Fecha: _ _/_ _/_ _ _ _ (dd/mm/aaaa) Fecha: _ _/_ _/_ _ _ _(dd/mm/aaaa)
