# Supplementary material for: Effectiveness of a virtual intervention for primary healthcare professionals aimed at improving attitudes towards the empowerment of patients with chronic diseases: study protocol for a cluster randomized controlled trial (e-MPODERA project)
Source: Trials. 2017 Oct 30;18:505. doi: 10.1186/s13063-017-2232-9 (PMC5663036; doi:10.1186/s13063-017-2232-9)
Supplement: Supplementary file 4 — Patient-Practitioner Orientation Scale (PPOS) questionnaire. (DOCX 16 kb) [file 13063_2017_2232_MOESM4_ESM.docx]

**Additional file 4: Patient-Practitioner Orientation Scale (PPOS) questionnaire**

Las afirmaciones que se presentan a continuación se refieren a las creencias que las personas podrían tener en relación con los profesionales sanitarios (médicos, enfermeras…), los pacientes y la atención médica. Por favor, lea cada frase y marque el número que indique el nivel de acuerdo o desacuerdo que usted tiene en cada caso.

| **1** | **2** | **3** | **4** | **5** | **6** |
| --- | --- | --- | --- | --- | --- |
| **Fuertemente en desacuerdo** | **Moderadamente en desacuerdo** | **Ligeramente en desacuerdo** | **Ligeramente de acuerdo** | **Moderadamente de acuerdo** | **Fuertemente de acuerdo** |

| 1. El profesional sanitario es quien debería decidir lo que se habla durante la consulta | 1 2 3 4 5 6 |
| --- | --- |
| 1. Aunque hoy en día la atención sanitaria es menos personal, este es un pequeño precio a pagar por los avances médicos | 1 2 3 4 5 6 |
| 1. La parte más importante de la consulta médica habitual es la exploración física | 1 2 3 4 5 6 |
| 1. A menudo es mejor para los pacientes que no tengan una explicación completa de su condición médica | 1 2 3 4 5 6 |
| 1. Los pacientes deberían confiar en el conocimiento de sus médicos y no intentar averiguar sobre su condición de salud por su cuenta | 1 2 3 4 5 6 |
| 1. Cuando los médicos hacen muchas preguntas sobre los antecedentes del paciente, se están entrometiendo demasiado en asuntos personales | 1 2 3 4 5 6 |
| 1. Si los médicos son realmente buenos en el diagnóstico y tratamiento, la forma en la que se relacionan con los pacientes no es tan importante | 1 2 3 4 5 6 |
| 1. Muchos pacientes continúan haciendo preguntas a pesar de que no están aprendiendo nada nuevo | 1 2 3 4 5 6 |
| 1. Los pacientes deberían ser tratados como si fueran compañeros del médico, iguales en poder y estatus | 1 2 3 4 5 6 |
| 1. Los pacientes generalmente quieren noticias tranquilizadoras en lugar de información sobre su salud | 1 2 3 4 5 6 |
| 1. Si las principales herramientas de un profesional sanitario son mostrarse abierto y cálido, no tendrá mucho éxito | 1 2 3 4 5 6 |
| 1. Cuando los pacientes están en desacuerdo con su médico, esto es una señal de que el médico no tiene el respeto y la confianza del paciente | 1 2 3 4 5 6 |
| 1. Un plan de tratamiento no puede tener éxito si está en conflicto con el estilo de vida o los valores del paciente | 1 2 3 4 5 6 |
| 1. La mayoría de los pacientes quieren entrar y salir de la consulta del médico lo antes posible | 1 2 3 4 5 6 |
| 1. El paciente siempre debe saber que el médico es el responsable | 1 2 3 4 5 6 |
| 1. No es tan importante conocer la cultura y antecedentes del paciente para tratar su enfermedad | 1 2 3 4 5 6 |
| 1. El humor es un ingrediente importante en el tratamiento médico del paciente | 1 2 3 4 5 6 |
| 1. Cuando los pacientes buscan información médica por su cuenta, esto generalmente confunde más que ayuda | 1 2 3 4 5 6 |
