# Supplementary material for: Effectiveness of a virtual intervention for primary healthcare professionals aimed at improving attitudes towards the empowerment of patients with chronic diseases: study protocol for a cluster randomized controlled trial (e-MPODERA project)
Source: Trials. 2017 Oct 30;18:505. doi: 10.1186/s13063-017-2232-9 (PMC5663036; doi:10.1186/s13063-017-2232-9)
Supplement: Supplementary file 5 — Patient Activation Measure (PAM) questionnaire. (DOCX 16 kb) [file 13063_2017_2232_MOESM5_ESM.docx]

**Additional file 5: Patient Activation Measure (PAM) questionnaire**

(Insignia Health PAM^®^ Survey <http://www.insigniahealth.com/products/pam-survey>)

A continuación se presentan algunas afirmaciones que a veces las personas hacen cuando hablan de su salud.

Por favor, indique qué tan de acuerdo o en desacuerdo está usted con cada declaración. Para puntuar las opciones, observe el cuadro que le mostramos a continuación y marque del 1 al 4 donde proceda. También puede marcar N/A si la afirmación no es aplicable a su caso. Por favor, conteste de forma sincera, sin pensar en lo que los demás esperarían que dijera.

| **1** | **2** | **3** | **4** | **N/A** |
| --- | --- | --- | --- | --- |
| **Fuertemente en desacuerdo** | **En desacuerdo** | **De acuerdo** | **Fuertemente de acuerdo** | **No aplicable a mi caso** |

| 1. Cuando todo está dicho y hecho, yo soy la persona responsable de cuidar de mi salud | 1 2 3 4 N/A |
| --- | --- |
| 1. Tener un rol activo en mi propio cuidado es el factor más importante que afecta a mi salud | 1 2 3 4 N/A |
| 1. Estoy seguro/a de que puedo ayudar a prevenir o reducir problemas asociados a mi condición de salud | 1 2 3 4 N/A |
| 1. Conozco cómo actúa cada uno de los medicamentos que me han prescrito | 1 2 3 4 N/A |
| 1. Estoy seguro/a de que puedo decir si necesito ir al médico o si puedo cuidar de mi problema de salud por mí mismo/a | 1 2 3 4 N/A |
| 1. Estoy seguro/a de que puedo decirle a mi médico cuáles son mis preocupaciones, aunque no me haya preguntado por ellas | 1 2 3 4 N/A |
| 1. Estoy seguro/a de que soy capaz de seguir en casa el tratamiento médico que pudiera necesitar | 1 2 3 4 N/A |
| 1. Entiendo mis problemas de salud y qué los causan | 1 2 3 4 N/A |
| 1. Conozco qué tratamientos están disponibles para mis problemas de salud | 1 2 3 4 N/A |
| 1. He sido capaz de mantener los cambios de estilo de vida, como comer adecuadamente o hacer ejercicio | 1 2 3 4 N/A |
| 1. Sé cómo prevenir problemas relacionados con mi salud | 1 2 3 4 N/A |
| 1. Estoy seguro/a de que puedo encontrar soluciones cuando surjan nuevos problemas relacionados con mi salud | 1 2 3 4 N/A |
| 1. Estoy seguro/a de que puedo mantener los cambios en mi estilo de vida, como comer adecuadamente y hacer ejercicio, incluso durante momentos de estrés | 1 2 3 4 N/A |
